# Incorporation of robotic automated transcranial Doppler to screen for patent foramen ovale (PFO) and quantify right-to-left shunt severity in the evaluation of ischemic stroke patients for etiology and PFO management

**DOI:** 10.3389/fneur.2024.1481817

**Published:** 2025-02-13

**Authors:** Ira Chang, Yasaman Pirahanchi, Simon Izaguirre, Richard Rodriguez, Alyssa Wicknick

**Affiliations:** Swedish Medical Center, Englewood, CO, United States

**Keywords:** robotic automated transcranial Doppler, patent foramen ovale, right to left shunt, ischemic stroke, transesophageal echocardiography, transthoracic echocardiography

## Abstract

**Background:**

Right-to-left shunt (RLS) associated with patent foramen ovale (PFO) is common among cryptogenic strokes. Current diagnostic tools have limitations. Transthoratic echocardiography (TTE) is not as sensitive as Transesophageal echocardiography (TEE), TEE is invasive, and manual transcranial Doppler (TCD) requires trained staff to operate. Robotic automated TCD (raTCD) may be feasible and comparable to manual TCD. The study's purpose was to determine the rate of RLS detection using raTCD and combine the Spencer Logarithmic Scale (SLS) with the Risk of Paradoxical Embolism (RoPE) to identify patients at risk of PFO associated stroke or TIA.

**Methods:**

This single-center retrospective cohort study included adult patients (≥18 y/o) admitted from December 2021 to December 2022 with a stroke or transient ischemic attack. Those with no bone window or stroke mimics were excluded. Patients with an RLS on raTCD received a second scan at the physician's discretion. The SLS combined with the RoPE score was used to generate a modified screening PFO-Associated Stroke Causal Likelihood (msPASCAL) classification.

**Results:**

Of 212 patients who received raTCD, the mean age was 56, 14% were >65 years old, most were white (72%), predominantly male (59%), 52% had cryptogenic strokes, and 59% had an RLS. Most patients were able to perform Valsalva (89%) during raTCD. Of those with an RLS, 56% had an SLS of 1–2, while 44% had an SLS of 3–5. There were no significant differences in characteristics by SLS. Most patients with SLS grades 1–2 were classified using msPASCAL as unlikely to have PFO as stroke etiology (*n* = 55, 44%). A small number of large SLS grades 3–5 were considered probable for having a PFO-associated stroke while the rest were classified as possible (*n* = 38, 30.4%). Eight patients with positive RLS on raTCD had a negative TTE with bubbles; most of those had small RLS on raTCD (*n* = 5, 63%) or could not Valsalva due to sedation ((*n* = 6, 75%).

**Discussion:**

This study supports the feasibility of utilizing raTCD for RLS detection. The modified screening PASCAL classification can be generated for RLS patients and may be used to guide subsequent evaluation and management.

## Introduction

Patent foramen ovale (PFO) is an important cause of stroke, with higher rates of PFO detected in patients aged 18–60 (1). Among cryptogenic strokes, PFO has been identified in up to 50% of patients as a potential etiology (2). Having a large PFO has been identified as an important structural criterion for cryptogenic stroke causality, which can be identified using right-to-left shunt (RLS) severity scores (3). Even in non-cryptogenic strokes, a large RLS with a curtain pattern has been associated with a higher risk of stroke occurrence (4). However, ~25% of adults have a PFO, but only a fraction of these patients have a stroke (5). Many patients who have PFO and have had a stroke or transient ischemic attack (TIA) have other stroke risk factors such as atrial fibrillation (AF), large or small vessel disease, or cardiac valvular disease. In one study of asymptomatic individuals with and without PFO, there was no increased risk of stroke associated with PFO (6). Even if the overall percentage of stroke recurrence is low, a significant number of patients with cryptogenic stroke, or more specifically, embolic stroke of an undetermined source, are at risk for stroke recurrence, which can be devastating in young patients (5).

Stroke prevention in cryptogenic stroke patients consists of medical management with antiplatelets, anticoagulation, or percutaneous closure using septal occlusive devices. Mojadidi et al. summarized the five major trials that have compared medical management to PFO device closure with the primary outcome of stroke recurrence (7). Earlier results were equivocal, but a more recent study found a reduced rate of stroke recurrence after PFO device closure in patients with high-risk features (8). Saver et al. reported that among patients aged 18–60, those with no other apparent cause of stroke had the lowest rate of recurrent stroke after PFO device closure when compared to medical treatment (9). Patients with a Risk of Paradoxical Embolism (RoPE) score > 7 and a large PFO or atrial septal aneurysm are more likely to benefit most from PFO closure by reducing recurrent stroke risk based on a pooled analysis of the major PFO closure trial data (10). Because of this, identification of not only PFO but RLS size is of the utmost importance to potentially reduce the rate of stroke recurrence.

Transesophageal echocardiogram (TEE) is the gold standard for PFO identification, but transthoracic echocardiography (TTE) and transcranial Doppler (TCD) with bubble injection are also used, each having distinct advantages and disadvantages. TCD with bubble injection is more sensitive but less specific than TTE and TEE with bubble studies (11). The RLS severity can be quantified with TCD with bubble injection by using the Spencer Logarithmic Grading Scale (SLS), which counts microembolic signals (MESs) from 0 to 5, to correlate with RLS severity (12). TEE with bubble injection is currently the gold standard for intracardiac RLS but it is more invasive, often requires sedation, and is not as readily available since it requires a trained cardiologist to perform the procedure. Most patients who are sedated during TEE tolerate placement of the esophageal ultrasound probe; therefore, they cannot adequately perform a Valsalva maneuver which increases right-to-left bubble shunting with PFO, leading to underdiagnosis of PFO (13). TCD has reported sensitivities ranging from 91% to 100% and specificities ranging from 78% to 100% (14–22) TCD has fewer false negatives in studies compared to TTE or TEE, with ranges from 0% to 13%; TCD has a PPV range of 98% to 100%, with the highest PPV occurring when the High Intensity Transient Signals (HITS) occur in less than 9 s (14–24).

Tobe et al. conducted a comparative study between TCD and gold-standard TEE for the detection of RLS and examined the prognostication of recurrent stroke based on TCD shunt grade (25). Of the 284 patients who received both TCD and TEE, the group observed that TEE missed 43 (15.1%) of the shunts that were detected by TCD. Notably, 41.7% of those shunts missed by TEE were Spencer grade 3 or higher. Furthermore, younger patients exhibiting shunt grades 3 or higher through TCD examination were more likely to have suffered a TIA or other secondary event at follow-up. This underscores the assertion that shunt grade determined by TCD can be a strong predictor of the occurrence of TIA or stroke, complementing the predictive capacity of shunt detection by TEE alone.

Another study utilized TCD after bubble injection to correlate with increased stroke causality using the SLS. They found the optimal MES threshold for the diagnosis of a PFO-attributable stroke, which was also confirmed by TEE, was 46 MESs during the Valsalva maneuver (15). More recently, RLS testing using robotic automated AI software enhanced TCD (raTCD) identified three times more cases than TTE (26). Among patients who also received raTCD and TEE, 86% had an RLS diagnosed on raTCD, compared to 57% of patients found to have PFO using TEE with bubbles (26). TCD, however, is not as specific as TEE for detecting PFO since extracardiac causes of RLS, primarily pulmonary arteriovenous malformations, are also detected. These are typically very small and can be distinguished on TCD by the timing of bubbles appearing on the TCD recording after bubble injection, which is longer due to extra transit time via extracardiac shunts (27). In terms of identifying stroke etiology, the increased sensitivity of TCD to extracardiac shunting as a cause of stroke is advantageous.

Based on this background, the raTCD system was incorporated into a new stroke evaluation algorithm for RLS screening in young patients who present with stroke or TIA at our comprehensive stroke center. The purpose of this retrospective review was to review the RLS occurrence and severity distribution in our stroke and TIA patients using the SLS, the PFO-Associated Stroke Causal Likelihood (PASCAL) score, the RoPE score, and a SLS that is specific to TCD by combining it with the RoPE score for each patient to generate a modified screening PASCAL (msPASCAL) classification. Patients who had a mismatch between their raTCD result and subsequent diagnostic examination (TTE or TEE) were reviewed in detail.

## Materials and methods

### Study design

This was a retrospective cohort study at a Comprehensive Stroke Center in Colorado. The local institutional review board approved the study with a waiver of patient informed consent, and Health Insurance Portability and Accountability Act authorization. Deidentified data may be shared by the principal investigator upon reasonable request. Artificial intelligence using the ChatGPT-3.5 Turbo model was used to assist with grammar and sentence flow. This study included all consecutively admitted patients with a diagnosis of acute ischemic stroke (AIS) or TIA from December 2021 to December 2022 who received raTCD. Patients with ages < 18, those who had no bone window present, or who were diagnosed with a stroke mimic were excluded from the study.

All clinical data were manually abstracted from the electronic medical record. These data included patient age on hospital admission, sex (male and female), patient-reported race (white, Black, Hispanic, Asian, and Other), Trial of Org 10172 in Acute Stroke Treatment (TOAST) category (cardioembolic, large artery atherosclerosis, small vessel occlusion, other, and cryptogenic), cortical infarct on initial head imaging, ability to Valsalva, presence of bone window (yes/no), SLS (0, 1–2, 3–5), and RoPE score (28).

### Stroke evaluation workflow

**Figure 1:**
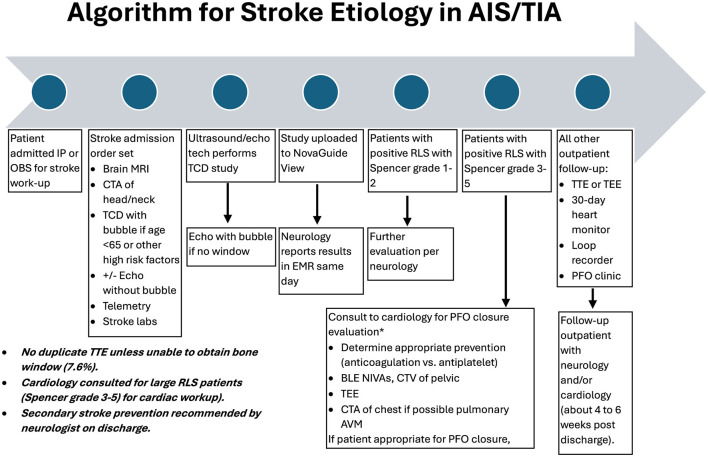
Algorithm for stroke etiology in acute ischemic stroke (AIS) and transient ischemic attack (TIA).

All patients admitted with AIS or TIA have a standard evaluation for stroke causality as indicated in the following algorithm (Figure 1). All adult patients aged 18–65 had raTCD with a bubble injection performed. If an SLS 3–5 RLS was found, cardiology was consulted, and the patient was usually referred for outpatient Holter monitoring and TEE in most cases. Occasionally, the patient was evaluated in the hospital if the risk of stroke recurrence was thought to be high. Patients aged >65 years old were considered for raTCD at the treating physician's discretion (*n* = 31, 13% in this study), usually in cases of venous thrombosis and suspected hypercoagulable state due to malignancy. These data were included in the review population.

### raTCD testing

NovaGuide 2 Intelligent Ultrasound, a raTCD system comprised of two megahertz bilateral probes on a headset that has five degrees of freedom within the robotic mechanism, was used to perform the TCD with saline bubble injections to detect MESs recorded in the middle cerebral artery (MCA). Data were uploaded to the hospital's picture archiving and communication system and NovaGuide View, a cloud-based data streaming and reporting application platform that could be accessed by the study readers. Both are manufactured by NeuraSignal, Inc., Dba NovaSignal, in Los Angeles, California.

Ultrasound and echocardiography technologists were trained to perform this test at the bedside. The patient is placed in a supine position up to 45 degrees of elevation, and their head is placed into the head-stabilizing cradle, which supports bilateral robotic probes. Using specially designed fiducials to engage the probe software and mark a region to search using AI software to locate the best transtemporal window, the NovaGuide autonomously located the vasculature and performed MCA signal optimization using M-mode and spectrogram to ensure the identification and record of the MCA signals bilaterally and continuously. While there is a manual probe that could have been used to find alternatives to temporal bone windows, these non-manual TCD operators were not trained in these techniques for ease of use and reproducibility.

Once the MCA signals were being recorded, a bedside nurse administered the bubble injection into a large bore intravenous catheter, using bacteriostatic saline and the drawback of blood into the syringe for agitation. Each patient received a bubble injection at rest and during a Valsalva strain, which was non-calibrated, calibrated with a tube, or induced by suctioning or abdominal pressure in mechanically ventilated patients. Patients who were unable to perform the Valsalva strain due to weakness or difficulty with comprehension attempted a calibrated effort by blowing into a plastic tube. Adequate Valsalva effort was checked for typical waveform changes by the technician. If the effort was inadequate, this was noted on the report, and the patient was referred for a follow-up study. If a patient had received a Definity contrast agent for TTE prior to raTCD, the test was delayed by 24 h. If a patient did not have any detectable vascular signals bilaterally, the bubble injection was not performed; therefore, the raTCD examination was not completed, and they were excluded from the study population. References to raTCD, TTE, and TEE in the remainder of this study were all performed with bubble injection.

### Bubble study interpretation

The SLS (0–5) was used to define the severity of RLS for patients with raTCD after a bubble injection (16). With SLS, grade 3 (30–100 bubbles) and higher was considered a large RLS; grades 1–2 were small. The raTCD and bubble injection studies were interpreted by trained neurologists who accessed the web-based NovaGuide View application to view video clips with audio of the baseline MCA M-Doppler signals at rest and for 1 min after each bubble injection. Bubbles were counted by the device software with confirmation by a trained neurologist. The RLS grade severity was quantified using the SLS of 0 to 5 for a standard defined number of MESs that are counted bilaterally during each 1-min segment. If only a unilateral MCA signal could be found, bubble counts would be doubled. Even if patients had weak MCA signals bilaterally, bubbles can be seen after injection since they are very echogenic.

### Stroke classification

Stroke etiology was classified according to the TOAST criteria using diagnostic test results completed at the time of hospital discharge. Categories in the TOAST criteria are the following: cardioembolic, large artery atherosclerosis, small vessel occlusion, other determined etiology, and cryptogenic etiology. Patients with a cryptogenic stroke etiology could have multiple equally plausible explanations for their stroke (28).

Two scoring methods evaluating the risk of stroke caused by PFO have been created: the RoPE and the PASCAL score (10, 29, 30). The RoPE score uses clinical features such as comorbidities and age to calculate a score describing the likelihood of stroke caused by an identified PFO (29). The PASCAL combines the RoPE score with structural features of PFO, such as large shunt size or atrial septal aneurysm identified on TEE, into a classification system that indicates how likely the stroke was caused by an identified PFO: unlikely, possible, or probable (10). Since TEE is not typically obtained during the inpatient workup at our comprehensive stroke center, an alternative novel approach using our raTCD results to screen and substitute for the PFO structural size feature of the standard PASCAL classification system was developed.

At our center, patients who had a positive RLS detected by raTCD were categorized into two groups based on shunt size: small shunts (SLS Grade 1–2), and large shunts (SLS 3–5). These raTCD-derived measures of RLS size were used in combination with the RoPE score to classify each patient with a msPASCAL (Table 1). Correspondingly, patients with RoPE of < 7 and SLS of 1–2 were classified as “unlikely” to have a PFO-associated stroke or TIA. Those with a RoPE of < 7 and a SLS shunt grade of 3–5 or a RoPE score of ≥7 and an SLS shunt grades 1–2 were both classified as msPASCAL of “Possible” for PFO-associated stroke causality. Patients with a RoPE of ≥7 and an SLS shunt grade of 3–5 were msPASCAL of “Probable” and most likely to have a PFO-associated stroke cause.

**Table 1:** msPASCAL classification scheme.

**RoPE Score**	**SLS 1-2**	**SLS 3-5**
< 7	Unlikely	Possible
≥7	Possible	Probable

RoPE, Risk of Paradoxical Embolism; msPASCAL, modified screening PFO-Associated Stroke Causal Likelihood (msPASCAL); SLS, Spencer Logarithmic Scale.

### Statistical analysis

Data were summarized with means and standard deviations, or proportion (count) as appropriate. *T*-tests and analysis of variance tests compared continuous measures between groups, while chi-square and Fisher's exact tests compared proportions between groups. All analyses were conducted using SAS 9.4 (Cary, NC). Patient characteristics were compared by SLS, and a diagnostic test was received. An alpha of 0.05 was used to define s tatistical differences for all comparable analyses.

## Results

**Figure 2:**
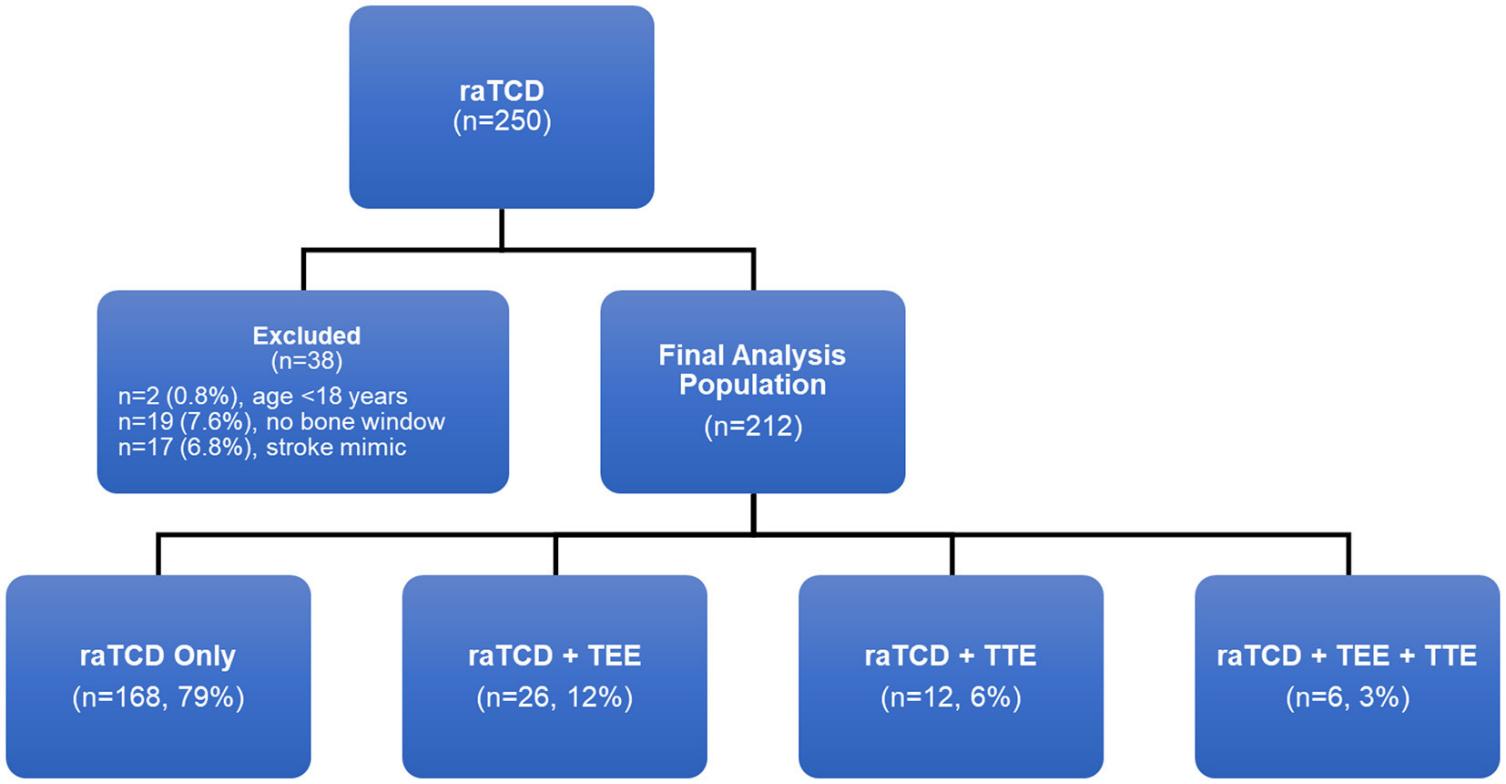
Patient population flow chart.

There were 250 patients who received raTCD (Figure 2). Of those, 38 (15%) met one of the exclusion criteria and were not included in the final analysis set, including 19 (7.6%) who were excluded due to no bone window. In the remaining 212 patients in the analysis set, 79% were screened with raTCD only, and 12% had both raTCD and TEE.

### Study population demographics and raTCD RLS results

Overall, patients had a mean (SD) age of 55.8 (11.3) years (Table 2); 14% (*n* = 31) were older than 65 years old. Most were male and identified as white. The most prevalent TOAST category with 111 patients (52.4%) was cryptogenic strokes. A majority of patients (89%) were able to Valsalva adequately for their PFO imaging studies. According to raTCD, 59% (*n* = 125) of patients were identified as having RLS. Of those with an RLS, 56% (*n* = 70) had a SLS of 1–2, and 44% (*n* = 55) had a SLS of 3–5. There were no significant univariate differences in demographics or initial stroke characteristics between patients based on their raTCD SLS. Notably, there was no significant association between the ability to perform an adequate Valsalva maneuver and the raTCD SLS (*P* = 0.72).

**Table 2:** Study population demographics.

**Variable, *n* (%)**	**Overall (*n =* 212)**	**Spencer 0 (*n =* 87, 41%)**	**Spencer 1-2 (*n =* 70, 33%)**	**Spencer 3-5 (*n =* 55, 26%)**	**P**
**Age**, mean (SD), years	55.8 (11.3)	56.1 (10.5)	56.9 (11.1)	53.7 (12.7)	0.28
**Sex**					0.77
Female	87 (41.0%)	35 (40.2%)	31 (44.3%)	21 (38.2%)	
Male	125 (59.0%)	52 (59.8%)	39 (55.7%)	34 (61.8%)	
**Race**					0.97
Caucasian	152 (71.7%)	66 (75.9%)	50 (71.4%)	36 (65.5%)	
African American	10 (4.7%)	3 (3.4%)	4 (5.7%)	3 (5.5%)	
Hispanic	27 (12.7%)	9 (10.3%)	9 (12.9%)	9 (16.4%)	
Asian	7 (3.3%)	3 (3.4%)	2 (2.9%)	2 (3.6%)	
Other	16 (7.5%)	6 (6.9%)	5 (7.1%)	5 (9.1%)	
**TOAST category**					0.10
Cardioembolic	24 (11.3%)	11 (12.6%)	10 (14.3%)	3 (5.5%)	
Large artery atherosclerosis	37 (17.5%)	17 (19.5%)	15 (21.4%)	5 (9.1%)	
Other	21 (9.9%)	8 (9.2%)	9 (12.9%)	4 (7.3%)	
Small vessel occlusion	19 (9.0%)	11 (12.6%)	3 (4.3%)	5 (9.1%)	
Cryptogenic	111 (52.4%)	40 (46.0%)	33 (47.1%)	38 (69.1%)	
**Valsalva**					0.72
Able	189 (89.2%)	76 (87.4%)	64 (91.4%)	49 (89.1%)	
Not able	23 (10.8%)	11 (12.6%)	6 (8.6%)	6 (10.9%)	
Transthoracic echocardiogram	18 (8.5%)	7 (8.0%)	4 (5.7%)	7 (12.7%)	0.37
Transesophageal echocardiogram	32 (15.1%)	7 (8.0%)	7 (10.0%)	17 (30.9%)	**< 0.0001**

SD, standard deviation; TOAST, Trial of Org 10172 in acute stroke treatment. Bold *p*-values indicate statistically significant differences when compared by Spencer grades.

### Comparisons of patient characteristics by diagnostic test received raTCD, TTE, and TEE

Only patients with a positive raTCD (*n* = 125) received a second scan: 25% (*n* = 31) had a TEE, 10% (*n* = 13) had a TTE, and 4% (*n* = 5) received all three studies (Table 3). Age (*p* = 0.0002) and sex (*p* = 0.01) were significantly different when compared by the type of scans received. In a *post-hoc* analysis, patients who received the raTCD and TEE were significantly younger than those who received raTCD only (*p* < 0.0001) with no other statistically significant differences in age based on the type of scan received. In a *post-hoc* analysis of sex, patients who received raTCD and TTE were male significantly more often than patients who received raTCD and TEE (*p* = 0.02); in addition, patients who received raTCD only were male significantly more often than patients who received raTCD and TTE (*p* = 0.003). There were no other sex differences observed between groups, and there were no significant differences in ethnicity (*p* = 0.43). There was a trend (*p* = 0.09) toward a higher rate of cryptogenic strokes among patients who received a raTCD and a TEE when compared to other scan types. There was also a trend (*p* = 0.09) toward a lower rate of patients who were not able to Valsalva among patients who received only a raTCD when compared to other scan types. Patients who received a raTCD and TTE had the lowest rates for the ability to Valsalva, but there were no significant differences.

**Table 3:** Study population demographics by scan types.

**Variable, *n* (%)**	**raTCD Only (*n =* 168)**	**raTCD + TEE (*n =* 26)**	**raTCD + TTE (*n =* 13)**	**raTCD + TEE + TTE (*n =* 5)**	***P***
**Age**, mean (SD), years	57.3 (10.3)	47.3 (14.3)	55.2 (9.2)	51.0 (14.1)	**0.0002**
**Sex**					**0.01**
Female	64 (38.1%)	18 (69.2%)	4 (30.8%)	1 (20.0%)	
Male	104 (61.9%)	8 (30.8%)	9 (69.2%)	4 (80.0%)	
**Race**					0.43
Caucasian	125 (74.4%)	17 (65.4%)	8 (61.5%)	2 (40.0%)	
African American	9 (5.4%)	0 (0.0%)	0 (0.0%)	1 (20.0%)	
Hispanic	19 (11.3%)	5 (19.2%)	1 (15.4%)	1 (20.0%)	
Asian	4 (2.4%)	2 (7.7%)	1 (7.7%)	0 (0.0%)	
Other	11 (6.5%)	2 (7.7%)	2 (15.4%)	1 (20.0%)	
**TOAST category**					0.09
Cardioembolic	21 (12.5%)	1 (3.8%)	1 (7.7%)	1 (20.0%)	
Large artery atherosclerosis	32 (19.0%)	2 (7.7%)	3 (23.1%)	0 (0.0%)	
Other	18 (10.7%)	1 (3.8%)	2 (15.4%)	0 (0.0%)	
Small vessel occlusion	16 (9.5%)	1 (3.8%)	0 (0.0%)	2 (40.0%)	
Undetermined	81 (48.2%)	21 (80.8%)	7 (58.3%)	2 (40.0%)	
**Valsalva**					0.09
Able	153 (91.1%)	23 (88.5%)	9 (69.2%)	4 (80.0%)	
Not able	15 (8.9%)	3 (11.5%)	4 (30.8%)	1 (20.0%)	

raTCD, robotically assisted transcranial Doppler; TEE, transesophogeal echocardiogram; TTE, transthoracic echocardiogram; SD, standard deviation; TOAST, Trial of Org 10172 in acute stroke treatment. Bold *p*-values indicate statistically significant differences when compared by diagnostic tool utilized.

### Detailed description of raTCD and TTE results

In patients who had TTE and raTCD, none of the negative raTCD results had a corresponding positive TTE. In contrast, five out of 11 positive raTCD RLS had a negative TTE. A detailed review of these five patients with mismatched diagnostic results showed that the majority (*n* = 4) had small RLS on raTCD testing (Supplementary Table 1). The 5th patient had an SLS 3–5 with Valsalva, who subsequently had a positive TEE for larger PFO, despite a negative TTE.

### Detailed description of raTCD and TEE results

There were no patients who had a negative diagnostic raTCD and a positive TTE. There were eight negative TEE results in patients who had positive RLS detected by raTCD. In a detailed review of these eight patients with mismatched diagnostic results, most of the patients were unable to Valsalva on TEE (Supplementary Table 2). Five of the eight patients also had a small RLS detected by raTCD. Finally, six of the eight patients had cryptogenic strokes.

### msPASCAL score

Of all patients with a positive RLS (*n* = 125), 17 patients (14%) had both high-risk clinical and large RLS that placed them into a msPASCAL probable category (Table 4). Among the 53 patients (42%) with a msPASCAL score of possible categories, 15 had an SLS 1–2, and 38 had an SLS 3–5. This makes a total of 70 patients (56%) who were prioritized for expedited cardiology evaluation and appropriate interim medical management. The remaining 55 patients (44%) who had neither high-risk clinical nor structural features were placed in the unlikely category. In patients with cryptogenic stroke (*n* = 71), a higher percentage of probable PFO-associated causality was seen in 12 (17%) patients, and 33 patients (46%) were in the possible category, resulting in a total of 45 patients (63%) who possibly had a PFO-associated stroke referred for appropriate management (Table 5). The remaining 26 patients (37%) were classified as unlikely due to small PFO and low-risk clinical features.

**Table 4:** Modified screening PASCAL classification for all patient with RLS.

	**msPASCAL group**		
**Spencer grade**	**Unlikely (*****n** =* **55, 44%)**	**Possible (*****n** =* **53, 42%)**	**Probable (*****n** =* **17, 14%)**
1 to 2	55 (44.0%)	15 (12.0%)	0
3 to 5	0	38 (30.4%)	17 (14.0%)

msPASCAL, modified screening PFO-Associated Stroke Causal Likelihood.

**Table 5:** Modified screening PASCAL classification for all patient with RLS and cryptogenic strokes.

	**msPASCAL group**		
**Spencer grade**	**Unlikely (*****n** =* **26, 37%)**	**Possible (*****n** =* **33, 46%)**	**Probable (*****n** =* **12, 17%)**
1 to 2	26 (36.6%)	7 (9.9%)	0
3 to 5	0	26 (36.6%)	12 (16.9%)

msPASCAL, modified screening PFO-Associated Stroke Causal Likelihood.

## Discussion

This study was successful in examining the effect of incorporating raTCD into the standard stroke evaluation algorithm. Without a vascular lab, we obtained a TCD system for bubble injection several years ago, but there were limitations with technician staffing and neurology availability at the bedside, which was required for interpretation. Using raTCD, however, we were able to train ultrasound and echocardiogram technicians, as well as neurologists, without prior vascular-specific training (or certification), to perform and interpret these tests. This suggests good feasibility of adopting a wider range of hospital systems than manual TCD, which has required expert staffing in the past. We were able to screen for any RLS, not just intracardiac, and did find a significant source of stroke in a patient due to a pulmonary AVM (31). Also in this study, RoPE and PASCAL scores were combined to calculate a msPASCAL to classify the patients' likelihood of having a stroke or TIA caused by PFO using raTCD, which could potentially guide treatment without obtaining TTE or TEE. Patients who could benefit from more targeted medical management and expedited cardiology follow-up on discharge could be identified and treated sooner using raTCD and the msPASCAL, but further data are needed to confirm this.

### Bone window rate

An advantage of raTCD is its lower rate of failure in obtaining a bone window to visualize MCA flow signals compared to manual TCD, which ranges from 8% to 25% in multiple series (32). In a study directly comparing manual TCD performed by a registered vascular technologist to the raTCD system we used, the no-window rate was 4.1% vs. 3.5% with raTCD (33). In our study, 7.6% (*n* = 19) of patients in the initial dataset were excluded as they had no bone window to complete a bubble injection. This was performed by ultrasound and echocardiography technicians who were trained 1 week before going live at our institution. One difference that we should acknowledge is that there was a low proportion of African American and Asian races, who are recognized to have higher no bone window rates of 10%−15% (24, 34). This phenomenon is thought to be associated with variations in bone thickness, porosity around the acoustic windows, and the attenuation of ultrasound energy transmission (35). Thus, in places with a higher distribution of these ethnicities, the bone window rate using raTCD will likely be higher than ours.

### RLS detection

In our study, five patients whose RLS were not identified on TTE, a majority (*n* = 4/5) were small RLS, with only a few bubbles, which can easily be missed on TTE (Supplementary Table 1). Some of these cases could also have been small pulmonary AVM, but in most of these cases (3 out of 4), RLS was not thought to be the cause of stroke, so further identification of intracardiac RLS with TEE, which is thought to be better at identifying positive cases than TTE, was not pursued. Prior studies that did check for intracardiac shunting in all TCD-positive patients found specificity ranging from 86% to 92%, especially in high-grade RLS patients, since alternative non-cardiac causes are rare (16, 26). The fourth of these small RLSs were referred for outpatient TEE, and the results of the follow-up testing are unknown. There was one patient with a large RLS identified who was negative at rest on TTE, for which subsequent TEE demonstrated a large PFO, so the raTCD was accurate and the TTE was likely inaccurate. Since we did not have to wait for a TEE to be performed to determine a standard PASCAL score, which has been reported to take up to 21 days on average to perform, we were able to quickly screen patients more likely to have PFO as stroke etiology, guide secondary stroke prevention, and prioritize cardiology follow-up on discharge.

Similarly, there were several patients with RLS detected by raTCD who had concomitant negative TEE results (Supplementary Table 2). In reviewing these patients in detail, the majority (six out of eight) of patients did not have a Valsalva-associated bubble injection, which is required to conclude that a TEE bubble study is negative; therefore, we cannot conclude the absence of PFO (see more information in discussion section “Valsalva Effort” below). In only one of these eight TEE-negative patients was an alternative “provocative” maneuver to mimic what Valsalva attempted. All of these patients were referred for follow-up care and received in-hospital medical treatment for an RLS shunt seen on raTCD as the suspected cause of stroke, despite the negative TEE. The clinical utility of raTCD in the diagnosis of RLS as a cause of cryptogenic stroke or TIA and management is highlighted.

### Valsalva effort

PFO increases RLS and blood flow after the Valsalva strain maneuver. In patients who cannot perform an adequate Valsalva maneuver, such as aphasic or sedated patients, the bubble study is not conclusive for a negative RLS. Alternative methods can mimic the Valsalva strain, such as applying abdominal pressure, inducing coughing with suctioning for intubated patients, or using calibrated devices that the patient can blow into using a tube. The effectiveness of a strain-inducing maneuver can be measured by characteristic MCA waveform morphology dampening and mean velocity decrease of at least 25%, followed by an increase in amplitude above baseline after release. In our study, the patients' SLS grade was classified based on the resting bubble injection if they could not perform an adequate Valsalva, which likely resulted in an underestimated RLS positivity and severity. There was no difference in the ability of Valsalva when compared with SLS. It was found that patients with a positive raTCD and a negative TEE predominantly were unable to conduct the Valsalva strain maneuver during their TEE but were able to during their raTCD (Supplementary Table 2), which was potentially a source of the discordance between the two examinations.

### Shunt grade severity

Multiple studies have pointed to RLS size and other high-risk features on TEE that have been associated with increased risk of recurrent stroke and risk reduction with PFO closure (29, 36). In the CLOSE 2017 trial, patients who had an ASD or large interatrial shunt (>30 microbubbles in three cardiac cycles) and underwent PFO closure had a recurrent stroke rate of 1.4% in 3- to 6-year follow-up compared to 5.4% for the group that did not undergo PFO closure (29). In the REDUCE 2017 trial, moderate-to-large size PFO (6–25 or >25 microbubbles in three cardiac cycles) underwent PFO closure with a recurrent rate of 0% in a 3- to 6-year follow-up as compared to 6.3% for the group that did not undergo PFO closure (36). Because of these prior studies, we considered the finding of SLS of grades 3–5 on raTCD to be significant for clinical consideration of PFO-associated stroke and subsequent management. Lao et al. compared the SLS criteria to the International Consensus Criteria (ICC) for RLS detection and found that the SLS was more specific compared to ICC (specificity of 91.3% vs. 72.4%), with fewer false positive rates (7.7% vs. 24.4%), which they suspected was due to the higher number of bubbles required to define a large shunt by SLS (16).

### msPASCAL classification

Timely identification of PFO as a cause of stroke is important as it facilitates swift risk stratification, leading to appropriate clinical management at the time of discharge. This may involve escalating from aspirin to dual antiplatelet therapy or anticoagulation, as well as expediting cardiology follow-up for further workup and potential PFO closure (9, 37). However, diagnosis of PFO is challenging; the current gold-standard TEE is not standardly obtained during inpatient hospitalization, nor should it be in cases of severe stroke that should wait 1 to 2 months for clinical stability (38). Scheduling difficulties and the need for preliminary assessments such as Holter monitoring or insurance approval can prolong the process; raTCD presents an alternative screening tool to TEE for the early identification of patients with RLS. Since raTCD is less invasive in nature and rapidly accessible than TEE, we created the msPASCAL classification system to indicate which patients are more likely to have a stroke caused by PFO and who may benefit the most from PFO closure, as previously published by Kent using TEE (10). Our distribution of patients across the classification of unlikely, possible, and probable PFO was 44% (*n* = 55), 42% (*n* = 53), and 14% (*n* = 17), respectively. The msPASCAL score aligns with the standard PASCAL score in most cases. This modified classification may be useful for medical management, device closure, and interventional cardiology consultation prior to discharge; future research is needed to evaluate the effect of using the msPASCAL score to guide treatment. Patients exhibiting RLS with SLS 3–5 necessitate follow-up, irrespective of if the shunt is non-PFO, as in pulmonary AVM, even if asymptomatic or incidental, do require attention. The ease of use of raTCD proves invaluable in these scenarios and ensures comprehensive assessment, aiding in timely interventions and appropriate medical management for patients with various RLS beyond PFO.

### Other advantages of raTCD

TCD uniquely characterizes and quantifies embolization in extracardiac RLS, such as in pulmonary AVM that might not otherwise have been discovered with TTE or TEE. One case series of 219 patients with pulmonary AVM reported that 34% of patients experienced neurologic events such as stroke and brain abscess (39). We previously presented a case report on a 38-year-old man in our series who presented with a small occipital stroke and was found on raTCD to have an SLS 5 RLS at rest and with Valsalva. Subsequent TEE did not reveal any intracardiac defects, but cardiac magnetic resonance imaging and chest computed tomography angiography identified pulmonary AVM as the embolic source, leading to embolization treatment (31). In addition, raTCD has been shown to be more feasible to introduce than conventional manual TCD, which has not been widely adopted despite known superior sensitivity when compared to TTE and TEE, in part because of its reliance on expert vascular ultrasound technicians (26, 33). Furthermore, prior research found TCD to be more cost-effective than echocardiography (14, 40). Standard TTE without bubbles was maintained in our stroke evaluation algorithm to evaluate for other structural causes of stroke such as intracardiac thrombus, low ejection fracture, or valvular disease.

### Limitations

This study was limited by the small sample size and retrospective nature. Receiving multiple diagnostic scans was rare, and there were no patients who received a positive TTE or positive TEE who had a negative raTCD. As previously mentioned, prior studies have observed that African American and Asian patients have a higher rate of no bone window, which was used as an exclusion criterion for this study. The exclusion of patients with inadequate bone windows is also a limitation to the study as this is a limitation to raTCD; manual TCD can be performed through other windows, such as occipital or submandibular. This study was conducted at a comprehensive stroke center. These results may not be generalizable to other hospital residents in areas with differing demographics or lower-level stroke center designations. Cardiology referral for follow-up data was collected, but the data on PFO closure were limited. The counting system for bubbles could underestimate the true severity of RLS. The patients' SLS grade was classified based on the resting bubble injection if they could not perform an adequate Valsalva, which likely resulted in an underestimated RLS positivity and severity. Furthermore, patients were not further tracked to determine whether they suffered a recurrent stroke. Because only patients with positive results on raTCD were examined with other diagnostic tools, we do not know how many patients may have been missed on raTCD and would have been diagnosed on TEE or TTE, and test accuracy calculations were not conducted. Future studies may consider evaluating age differences in results for patients older than 65 years old; due to the small sample of patients older than 65 years old in this study, we were unable to stratify analyses.

## Conclusion

This study supports the feasibility of utilizing raTCD for RLS diagnosis and identified a new way to classify the possible likelihood of stroke caused by PFO without the need for TEE. Among patients with a positive RLS on raTCD, but a negative PFO on TEE, a majority were unable to Valsalva on TEE but were able to Valsalva for the raTCD. This highlights another reason why raTCD may be superior to TEE as TEE is invasive, requiring sedation, and limits the ability to conduct the Valsalva maneuver which is associated with more accurate diagnostic results. A msPASCAL classification was developed replacing TEE results with SLS identified on raTCD and incorporating that with RoPE scores to categorize patients based on the likelihood that their stroke was attributable to PFO. The msPASCAL score may be useful to direct cardiology referral for TEE and PFO closure, but further data are needed to confirm this. The extension of raTCD to outpatient testing could yield better Valsalva results and potentially lead to improved secondary stroke prevention through the larger TIA patient population.

## Data Availability

The raw data supporting the conclusions of this article will be made available by the authors, without undue reservation.
