# Supplementary material for: Incorporation of robotic automated transcranial Doppler to screen for patent foramen ovale (PFO) and quantify right-to-left shunt severity in the evaluation of ischemic stroke patients for etiology and PFO management
Source: Front Neurol. 2025 Feb 13;15:1481817. doi: 10.3389/fneur.2024.1481817 (PMC11866324; doi:10.3389/fneur.2024.1481817)
Supplement: Supplementary file 1 [file Table_1.docx]

| **Supplemental Table 1: raTCD vs TTE Mismatches** | | | | | | | | | | |
| --- | --- | --- | --- | --- | --- | --- | --- | --- | --- | --- |
| **Age Sex** | **Vessel Location** | **RoPE** | **Medical Mgmt** | **TOAST** | **Valsalva raTCD** | **Valsalva TTE** | **Spencer Grade** | **PASCAL** | **Sent for F/U** | **Neuro Impression** |
| 67 F | Right MCA, bilateral cerebellum | 6 | Eliquis | Cryptogenic | Unable | Able | 1-2 | Unlikely | Yes | Possible |
| 37 M | Right MCA | 8 | DAPT | Cryptogenic | Able | Able | 3-5 | Probable | Yes | Yes |
| 64 M | Right ICA | 5 | Heparin gtt to eliquis | LAA | Able | Unable | 1-2 | Unlikely | Yes | No |
| 50 M | Left PCA, SCA, right vertebral | 7 | DAPT | Other | Able | Able | 1-2 | Possible | Yes | No |
| 31 F | Left cerebellar, vertebral artery | 10 | Eliquis | Other | Able | Able | 1-2 | Possible | No | No |
| F/U: follow-up, F: female, M: male, MCA: middle cerebral artery, ICA: internal carotid artery, PCA: posterior cerebral artery, SCA: superior cerebellar artery, DAPT: dual antiplatelet therapy, LAA: Large artery atherosclerosis, RoPE: risk of paradoxical embolism, Mgmt: management, TOAST: Trial of Org 10172 in Acute Stroke Treatment, raTCD: robotic automated transcranial doppler, TTE: transthoracic echocardiogram, PASCAL: PFO associated stroke causal likelihood. | | | | | | | | | | |
