# Supplementary material for: Incorporation of robotic automated transcranial Doppler to screen for patent foramen ovale (PFO) and quantify right-to-left shunt severity in the evaluation of ischemic stroke patients for etiology and PFO management
Source: Front Neurol. 2025 Feb 13;15:1481817. doi: 10.3389/fneur.2024.1481817 (PMC11866324; doi:10.3389/fneur.2024.1481817)
Supplement: Supplementary file 2 [file Table_2.docx]

| **Supplemental Table 2: raTCD vs. TEE Mismatches** | | | | | | | | | | |
| --- | --- | --- | --- | --- | --- | --- | --- | --- | --- | --- |
| **Age Sex** | **Vessel Location** | **RoPE** | **Medical Mgmt** | **TOAST** | **Valsalva raTCD** | **Valsalva TEE** | **Spencer Grade** | **PASCAL** | **Sent for F/U** | **Neuro Impression** |
| 39 F | Left MCA | 10 | DAPT | LAA | Unable | Able | 1-2 | Possible | Yes | No |
| 62 F | Bilateral Right cerebellar | 4 | Lovenox, Plavix, Prednisone, Hydroxyurea | Cryptogenic | Able | Unable | 3-5 | Possible | Yes | Possible |
| 51 M | Right SCA, Basilar artery | 5 | ASA | Cardio-embolism | Able | Unable | 1-2 | Unlikely | Yes | No |
| 68 F | Left MCA, Left ICA | 3 | Eliquis | Cryptogenic | Able | Unable | 1-2 | Unlikely | Yes | No |
| 32 F | Right MCA | 10 | Eliquis | Cryptogenic | Able | Able | 3-5 | Probable | Yes | Yes |
| 60 M | Right MCA, ICA | 4 | ASA | Cryptogenic | Able | Unable | 3-5 | Possible | Yes | Possible |
| 29 M | Left MCA | 8 | ASA | Cryptogenic | Able | Unable | 1-2 | Possible | Yes | No |
| 48 F | Bilateral posterior vertebral | 6 | ASA | Cryptogenic | Able | Unable | 1-2 | Unlikely | Yes | Possible |
| F/U: Follow-up, F: female, M: male, MCA: middle cerebral artery, ICA: internal carotid artery, PCA: posterior cerebral artery, SCA: superior cerebellar artery, DAPT: dual antiplatelet therapy, LAA: Large artery atherosclerosis, RoPE: risk of paradoxical embolism, Mgmt: management, TOAST: Trial of Org 10172 in Acute Stroke Treatment, raTCD: robotic automated transcranial doppler, TTE: transthoracic echocardiogram, PASCAL: PFO associated stroke causal likelihood. | | | | | | | | | | |
